# Comparison of perceptions in Canada and USA regarding cannabis and edibles

**DOI:** 10.1186/s42238-023-00213-9

**Published:** 2024-01-03

**Authors:** Janet Music, Brian Sterling, Sylvain Charlebois, Christine Goedhart

**Affiliations:** 1 Faculty of Arts & Social Sciences, Dalhousie University, Halifax, NS Canada; 2 SCS Consulting, Toronto, ON Canada; 3 Agri-Food Analytics Lab, Dalhousie University, Halifax, NS Canada; 4 University of British Columbia, Vancouver, BC Canada

**Keywords:** Cannabis, Cannabis policy, Canada, Cannabis stigma

## Abstract

**Background:**

Canada took a national approach to recreational cannabis that resulted in official legalization on October 17, 2018. In the United States (US), the approach has been more piecemeal, with individual states passing their own laws regulating adult use. The objective of this study was to compare the two jurisdictions.

**Methods:**

Two exploratory, quantitative surveys were administered in May of 2021 in both Canada and the US. One thousand forty-seven Canadian and 1037 US residents (age 19 and older) were surveyed on approaches to and attendant regulations of consumer cannabis. Tests of significance were performed to analyze differences between two groups.

**Results:**

No statistically significant differences exist between the two countries in terms of cannabis legalization acceptance. Usage rates among adults was similar with 45% of Canadians and 42% of Americans confirming they consume cannabis. Respondents maintain that they intend to increase their usage, with edibles attracting a rising level of interest from consumers.

**Conclusions:**

Results suggest that public policy in both Canada and the USA needs to change to reflect rapidly evolving acceptance of cannabis products in North America to realize potential economic returns.

## Introduction

Cannabis is the most widely used illicit drug in the world (Degenhardt & Hall 2012). Its consumption in the past has often been associated with negative social and economic outcomes (Castellanos-Ryan et al. 2021; Hartman et al. 2017). It has been estimated that between 129 and 170 million people world-wide consume cannabis daily (Adinoff & Reiman 2019; Petranker et al. 2020). Enforcement of the illegal status of cannabis in jurisdictions has often had serious negative consequences, particularly among disadvantaged and/or racialized communities (Earp et al. 2021; Kemper et al. 2021). The need to remedy this inequity has been a key driver of efforts to legalize (or decriminalize) possession and use of cannabis (Aaronson & Rothschild-Elyassi 2021; Earp et al. 2021). Regardless, the task for policymakers and public health officials is to now navigate a myriad of possibilities resulting from the widespread increased acceptance and consumption of cannabis. The objective of this exploratory research is to compare attitudes on forms of legalized cannabis in two jurisdictions.

Canada was the first G7 country to make recreational cannabis legal nationally (Ross 2018). There are now 19 US states that have legalized recreational use, out of a total of 36 that have permitted legal sale and consumption for adult use. This report examines the data from a survey on attitudes and behaviors relating to cannabis. The analysis and findings evolved into 4 themes: acceptance and consumption, normalization, edibles, and education. These form the basis for the findings and discussion in this report.

The popularity of cannabis as a recreational drug is due to its ability to alter sensory perception, relieve pain, and cause elation and euphoria (Mullins 2021; Peng & Shahidi 2021). Many consumers prefer cannabis edibles to smoking because they do not wish to smoke, plus there is no smell or second-hand smoke (Charlebois et al. 2020). Other advantages of edibles include convenience, discreetness, longer-lasting effects, less intense onset, and their ability to aid relaxation (Doran & Papadopoulos 2019). Disadvantages of edibles include delay or unpredictability of effects and inconsistency of distribution of cannabinoids in the product (Giombi et al. 2017). Manufacturers have been conducting their own research and making efforts to address these undesirable traits through product development.

Municipal laws may affect consumption levels by specifying where cannabis products are sold and how easily they may be accessed by existing and future users. In research on municipal by-laws, greater accessibility has been linked to higher cannabis usage levels (Gagnon et al. 2022; Gourdet et al. 2021). Shop density, that is, the number of stores in a given area and store location were two important characteristics that affected consumption level (Brinkman & Mok-Lamme 2019; Németh & Ross 2014). In the US, greater cannabis shop densities have been linked to higher odds of regular cannabis usage (Freisthler & Gruenewald 2014; Morrison et al. 2014). High store density was also linked to increased frequency and earlier adoption of usage including vaping and edibles (Borodovsky et al. 2016).

The manner in which cannabis is ingested is not routinely measured in population-based surveys (Johnson et al. 2016). Consumable alternatives to inhaled cannabis (e.g., infused food, drinks, candies) are becoming increasingly popular. End-of-year data from Seattle-based cannabis analytics firm Headset shows that in 2020 sales of all cannabis edibles grew by 60% across seven state markets (California, Colorado, Massachusetts, Michigan, Nevada, Oregon, and Washington)—amounting to sales of $1.23 billion in 2020, up from $767 million in 2019 (Schaneman 2021). That notable accomplishment meant edibles outperformed the total US cannabis market, which grew a hefty 54% last year. According to Headset Co., edibles increased their overall market share from 10.6% in 2019 to 11.1% in 2020 (Schaneman 2021). Reports suggest that the US edibles market is expected to surpass $10 billion by 2025 (Brightfield Group 2021), which would require a compound annual growth rate over 50%. Unsurprising, cannabis and food companies are putting their efforts into developing new product types, including faster-acting and strain-specific edibles.

## Methodology

The survey was administered online during 2 weeks in May 2021. Using an approach consistent with similar studies on consumption and trends (Beardsworth & Bryman 2004; Redman 2020), the survey was distributed across both countries (in English and French for Canada). All data were collected through a third-party market research firm, Angus Reid, using its proprietary sample of voluntary, self-selected participants titled The Angus Reid Forum. The Canadian panel is comprised of a sample frame of approximately 1.3 million adults, while the U.S. panel is comprised of approximately 1 million adults. Both panels are managed by Angus Reid. The integrity of each panel is established through the size of the sample frame. Panel members are representative of the location population. Recruitment sources for both panels include social media and digital advertising, media partnerships, and private sector partners that benefit from research conducted with the panel. Digital and print advertising messages are used to attract both broad and targeted groups.

The data were secured for this study on the Qualtrics online survey platform. Informed consent was granted by participants when then agreed to an online consent form and continued with the survey. Those that did not consent to the survey closed their browsers. The sample was not representative of the broader population by demographics or by cannabis use. However, results were monitored regularly to help ensure broad representation of residents in both countries. This study was performed in line with the principles of the Declaration of Helsinki. Approval was granted on April 27, 2021, by the Dalhousie University’s Research Ethics Board, REB file #: 2021–5528.

This study was derived from a quantitative analysis of data from online surveys of those aged 19 and over, living in the USA or Canada for at least 12 months. The first part of the survey included questions broadly related to perceptions of cannabis and cannabis-infused edible products (beverages were included in edibles for this assessment). Respondents were asked if they favored legalization of cannabis in general, their feelings about being known for consuming cannabis, how often they consume cannabis, and for what purpose. We sought to understand if there are correlations between the two North American countries and to compare the Canadian results against previous surveys. The second section asked questions about cannabis edibles, including perceived risks and barriers associated with edibles generally. This part was intended to explore differing perceptions of risks regarding edibles, gaps in knowledge about cannabis and its effects, and on which resources consumers rely to mitigate the risks and address those gaps. In the last section, we asked demographic questions about age, sex, geographic location (state or province), and the respondent’s income and education level. These questions were designed to allow us to dive more deeply into specific population segments. The instrument was pre-tested with 50 respondents across Canada, using similar questions from surveys developed in 2017 (Charlebois et al. 2018) and in 2019 (Charlebois et al. 2020). Usability and readability were sound, as in previous years; therefore, questionnaire did not require any subsequent major changes.

Canadian regions surveyed were the Atlantic Provinces, Québec, Ontario, the Prairies, and British Columbia (BC). These locations were chosen to maximize regional and socioeconomic variability and to maintain similarity with previous consumer attitude surveys. The Canadian sample size of 1047 was considered statistically adequate to reflect the approximately 30 million adults 19 years of age and older; based on the sampling design, the margin of error was 3.3%, 19 times out of 20 (Singh & Masuku 2014). Respondents took an average of just over 5 min to complete the survey. The completion rate was 92%, which was relatively high for surveys of this type (LaRose & Tsai 2014). In the USA, we asked for the name of the state in which the respondent resided. The following states were included in the sample: Alabama, Arizona, Arkansas, California, Colorado, Connecticut, Florida, Georgia, Hawaii, Idaho, Illinois, Indiana, Iowa, Kansas, Kentucky, Louisiana, Maryland, Massachusetts, Michigan, Minnesota, Mississippi, Missouri, Nebraska, Nevada, New Hampshire, New Jersey, New Mexico, New York, North Carolina, Ohio, Pennsylvania, Rhode Island, South Carolina, South Dakota, Tennessee, Texas, Utah, Vermont, Virginia, Washington, Wisconsin. The American sample size of 1037 was statistically sufficient to reflect the adult population with a margin of error of 3.1%, 19 times out of 20 (Singh & Masuku 2014). As this was an exploratory study using two distinct samples, one for Canada and one for the United States, there are limited predictive and comparative statistics that can be performed. We have performed two-tailed *p*-tests of significance on the data and included in the results.

## Results

### Characteristics of participants

The socio-demographics characteristics are reported for both jurisdictions as presented in Table 1. For the US, 52.04% of participants were females and 47.96% were males, respectively. In Canada, 59.51% were female, while the male participants accounted for the remaining 40.49%. Respondents were divided in three age groups: aged 18–34, 35–64, 64 + years. Respondents in the US were aged 18–34 years (29.61%), and the groups aged 35–64 years (43.86%) and 64 + years (22.92%) were similarly well represented. In Canada, respondents were, similarly, aged 18–34 years (35.37%), and the groups aged 35–64 years (41.42%) and 65 + years (23.21%) were similarly well represented as well. Also presented in Table 1 are comparisons for the sociodemographic characteristic for the population of cannabis users by country (Health Canada 2021; Jeffers et al. 2021). The unweighted data presented in this exploratory study falls into an acceptable margin of error for most of the sociodemographic categories for participants with a few notable exceptions within location. However, given the exploratory nature of the data and the stated limitations presented below, sampling bias should be modest within the study.

**Table 1:**
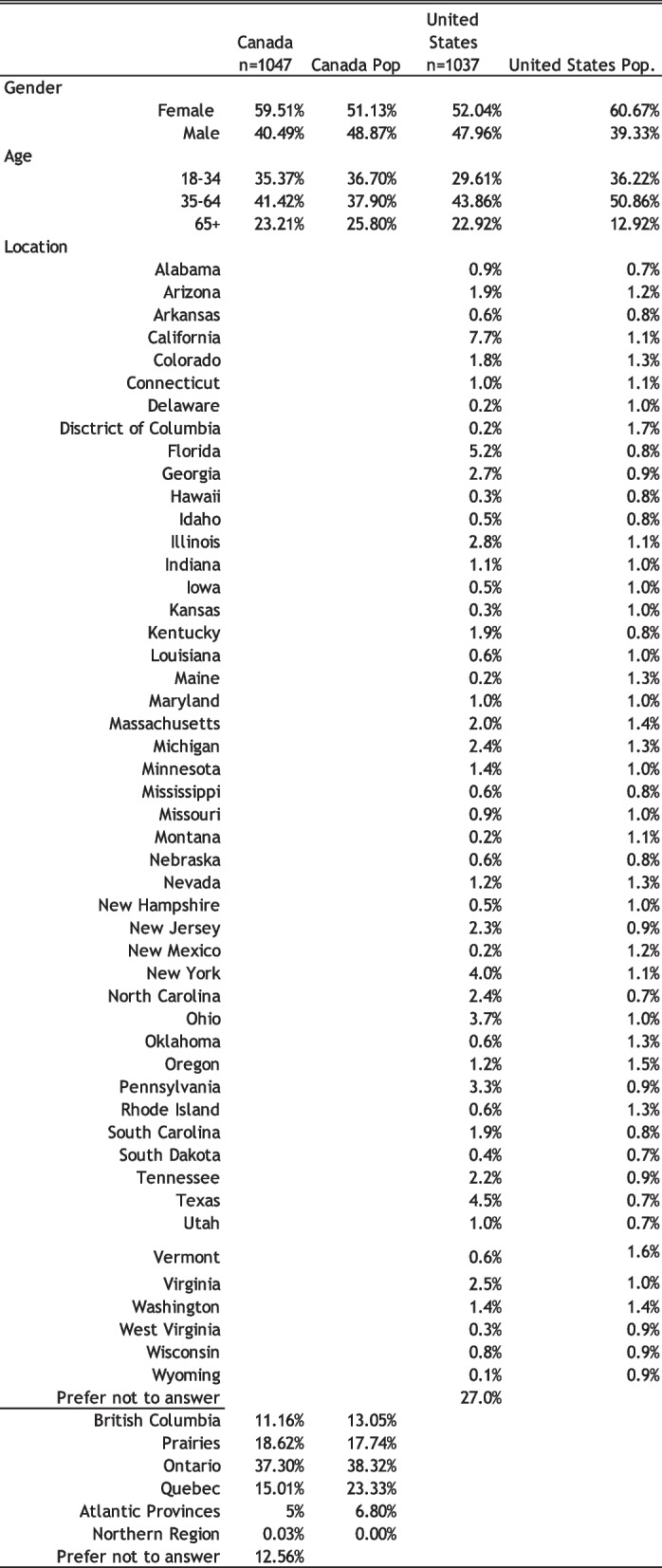
Side by side comparison of sample characteristics with unweighted population data of cannabis users, Canada and United States

### Acceptance and consumption

Regarding frequency of cannabis consumption, roughly 1 in 3 Americans who do consume reported they do so daily. In Canada, that portion was 25%. This difference was statistically significant (*p* < 0.001) Also of note was that 62% of US consumers reported they take cannabis at least once a week, while significantly, only 49% of Canadian respondents (*p* < 0.001) consume that frequently.

When asked directly if they support legalization of cannabis, a high percentage of Canadian and US respondents reported they do. Almost 8 in 10 Canadians (78%), and 3 of 4 Americans (75%), reported that they agree/strongly agree with legalization for recreational (adult-use) purposes. There was no statistically significant difference between these countries when it comes to acceptance.

**Fig. 1:**
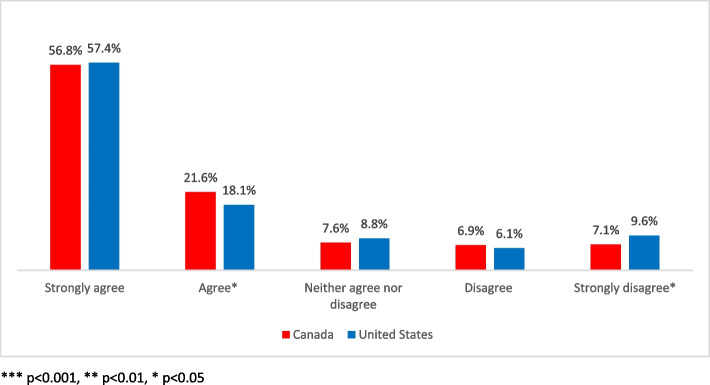
Current support of legalized cannabis: Canada and United States

Similarly, disagreement with legalization has declined to just under 16% in the USA and 14% in Canada (Fig. 1). Once again, no significant difference was present. Clearly, in both nations, the public has progressed beyond the tipping point and broadly accepts legal cannabis. However, that does not mean that a majority consumes cannabis or that illicit sources of cannabis are no longer a factor in purchasing behaviors.

**Fig. 2:**
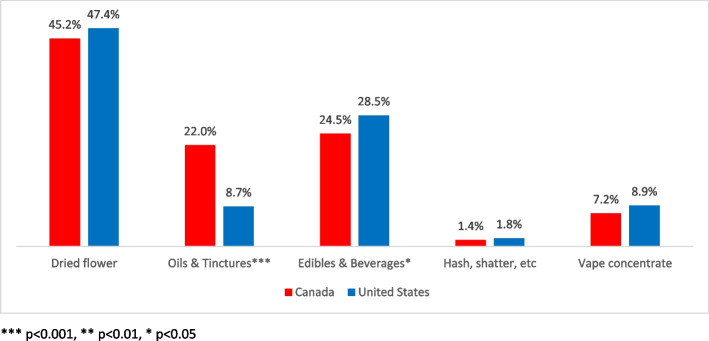
Popularity of cannabis formats in North America

In terms of types of cannabis purchased, dried flower (bud) remains the market share leader in both nations (Fig. 2), with Canadian cannabis consumers preferring it marginally less (45%) than their American counterparts (47%).

**Fig. 3:**
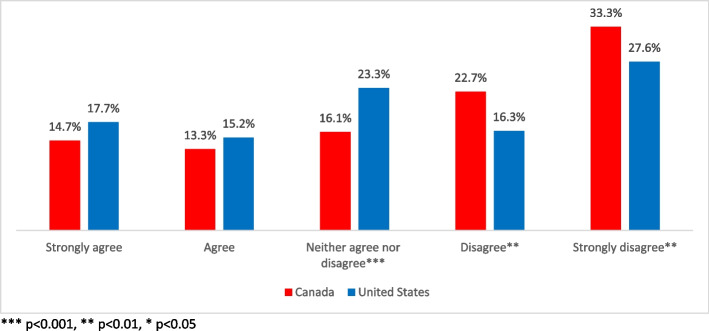
Support for municipal regulation of cannabis sellers

Indicative of possible future purchasing behavior, a substantial portion of respondents from both nations reported that municipalities should not be allowed to ban cannabis retail stores within their boundaries (Fig. 3). A majority of Canadian respondents (56%) agree or strongly agree that cities and towns should not be allowed to ban retailers; this was an almost total reversal of sentiment prior to legalization. Significantly, (*p* > 0.001) in the USA, 44% of respondents feel municipalities should not be permitted to ban cannabis stores.

In regard to the impact of COVID-19 on cannabis consumption, we uncovered measurable changes directly attributable to the pandemic. For example, just under one third of cannabis consumers in both countries reported they increased their intake of cannabis products between April 2020 and April 2021. When asked specifically about the impact of COVID-19, there was no significant difference between the two countries, with 14% of Canadian and 16% of US cannabis users reporting they consumed more during the pandemic.

On both sides of the international border, dried flower (or bud) was the format of choice for cannabis buyers: 45% of consumers in Canada compared to 47% in the USA (Fig. 2). As the responses reveal, interest in edibles (including beverages) was moderate in both countries, with almost 25% of Canadian consumers and just over 28% of American consumers reporting they prefer edibles. Interestingly, a significant difference exists between Canadian cannabis consumers (28%) than American (21%) report they do not buy edibles (*p* < 0.00).

**Fig. 4:**
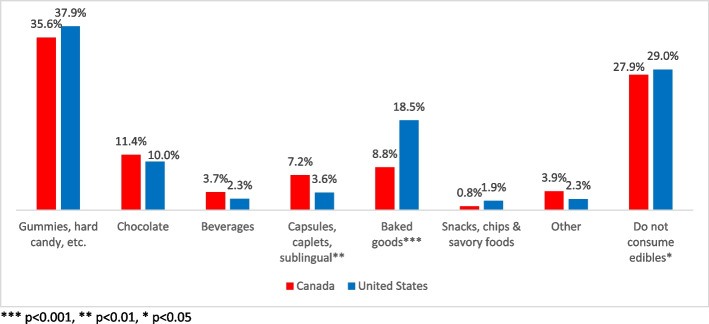
Preferred cannabis edible products—USA and Canada

Within the edible category, gummies and other sweet confections (e.g., mints, hard candies) lead the way in terms of popularity in both countries (Fig. 4). Chocolates were preferred almost equally by Canadian (12%) and American (10%) cannabis buyers. Looking at other types of edibles yielded a few statistically significant contrasts: for example, 22% of Canadian cannabis consumers reported they preferred oils and tinctures compared to only 9% in the USA (*p* < 0.001). Baked goods are preferred by 19% of American cannabis consumers versus just 9% of those in Canada (*p* < 0.001). A statistically higher number of Canadians (*p* < 0.001), roughly 63%, remain anxious about the risks that cannabis (and its edibles) represent to children and young adults, whereas 51% of Americans are concerned.

When asked directly, a modest portion of cannabis consumers reported they intend to increase their purchases of edibles in the future. Significantly, about 21% of American buyers reported they will, compared to 13% of Canadian consumers *p* < 0.001). A substantial minority (over 28%) in each country are uncertain, significant for both countries.

## Discussion

Beyond acceptance, consumption of recreational cannabis is growing. US cannabis consumers are less likely to purchase from legal sources than Canadians. This leaves significant cannabis purchases flowing through illegal (“legacy”) channels. It is possible that social behavior causes a slightly higher willingness by Canadians to buy from legal sources. However, a more likely factor is that recreational cannabis remains illicit in most states, and this may encourage a portion of recreational consumers to seek products from illicit sources. Establishing this would require a deeper examination of responses by state, comparing those from states where recreational (adult use) cannabis is legal with those where it is illegal.

In terms of types of products, dried flower still leads other formats and edibles are slowly gaining share. It is expected that, over time, the market for dried flower will stagnate and transition toward the edible market, as most health experts continue to discourage consumers from inhaling cannabis. Eating or drinking may also seem more “natural” to buyers.

The study’s findings challenge the notion that a country must legalize cannabis federally to change how it is broadly perceived socially. Canada and the USA offer two distinctive tales of cannabis legalization. Canadians are becoming comfortable with the idea of living where cannabis is legal in the entire country: social acceptance has markedly increased, stigmatization has declined, and knowledge of the science of cannabis is spreading, albeit slowly. Fewer Canadians express concern about others knowing they consume cannabis or care much if others partake than when cannabis was first legalized in October 2018. It will be intriguing to see how the market evolves in North America, along with public policy, as consumers become more knowledgeable about cannabis and its effects.

The distinctions between the countries may be shaped by cultural norms and are likely impacted by the current availability and breadth of choices in each country, which are influenced by many factors, including regulatory restrictions. For example, regarding social stigma, Canadians’ responses show they are more relaxed than their American neighbors about publicly acknowledging cannabis consumption. Aside from the higher level of acceptance noted above, more than half of Canadians reported municipalities should not be permitted to ban cannabis retailers within their boundaries. This is an almost complete reversal from observations in previous survey data (Charlebois et al. 2018, 2020). The portion of Americans reporting towns and cities should not be allowed to ban outlets is significantly lower.

This study presents a snapshot of the acceptance of cannabis in North America. While the results are interesting, there are some limitations that should be acknowledged. This exploratory study needs further research; thus, only high-level descriptive statistics are presented. Furthermore, the sample used in each country is not representative of the general population or the cannabis-using population. This study is an omnibus-style marketing research survey about cannabis and includes many aspects of usage that may appear to be disparate but are in fact interconnected by usage of cannabis itself. While percentages were on completers vs non-completers were provided but the third-party data collector, respondents who provided incomplete survey answers were not analyzed as stipulated in the informed consent at the beginning of the survey. If a respondent did not complete the survey, it was assumed, as directed by the questionnaire, that the participant revoked consent to collect their data. It is meant to be a holistic snapshot of perceived cannabis usage as self-reported by participants. Given the timeframe boundaries of some questions, respondents may over- or under-report usage (De Jong et al. 2015; Le et al. 2022; Palamar & Le 2022). Similarly, the timeframe straddles the global COVID-19 pandemic, which had profound impact on many people’s mental health and well-being (Graupensperger et al. 2021; Yıldırım & Arslan 2020). This may have influenced usage patterns and reporting for this survey. Regardless, this study offers an interesting snapshot of cannabis use in two countries. Further research on long-term usage and policy implication comparisons between the two countries would offer insights on usage. In addition, because these data were collected during a period of societal stress, these may not reflect typical usage patterns; therefore, future surveys should reassess these trends.

## Conclusion

Although trepidations and social stigma around cannabis use are declining, governments are aware that cannabis consumers and non-consumers alike remain concerned about public safety related to cannabis, particularly for youth and pets. This directly contradicts the broadly held acceptance of cannabis and the desire to see governments reduce their level of intervention in cannabis regulation. The data in this study reveal that North Americans generally feel cannabis is more accepted and less harmful than many imagined.

Consumers in North America now perceive cannabis in a more normalized way. It seems clear, based on views expressed by consumers, that cannabis sales will continue to increase and that edibles are positioned to capture an increasing share of the market. Yet, one of the roadblocks to further success, aside from regulatory restriction, is the level of knowledge and understanding most people have about cannabis. As shown in this report, there is a substantial desire (and need) for cannabis consumers and the “canna-curious” to learn more about the plant and how its phyto-chemicals may benefit their physical and mental health.

## Data Availability

The datasets used and/or analyzed during the current study are available from the corresponding author on reasonable request.

## Abbreviations

BC: British Columbia
Bud: Dried flower
COVID-19: Severe acute respiratory syndrome coronavirus 2 (SARS-CoV-2)
Legacy: Illegal channels
USA: United States of America
